# *14-3-3epsilon *contributes to tumour suppression in laryngeal carcinoma by affecting apoptosis and invasion

**DOI:** 10.1186/1471-2407-10-306

**Published:** 2010-06-19

**Authors:** Xing-Hua Che, Hong Chen, Zhen-Ming Xu, Chao Shang, Kai-Lai Sun, Wei-Neng Fu

**Affiliations:** 1 Department of Medical Genetics, China Medical University, 92 Beier Road, Heping District, Shenyang, 110001, China; 2 The E.N.T. Department, 463 Hospital of Chinese PLA, Shenyang, 110042, China; 3 Department of Neurobiology, China Medical University, 92 Beier Road, Heping District, ShengYang, 110001, China

## Abstract

**Background:**

14-3-3epsilon regulates a wide range of biological processes, including cell cycle control, proliferation, and apoptosis, and plays a significant role in neurogenesis and the formation of malignant tumours. However, the exact function and regulatory mechanism of 14-3-3epsilon in carcinogenesis have not been elucidated.

**Methods:**

The expression of *14-3-3epsilon *was assessed by RT-PCR and western blotting. The invasiveness and viability of Hep-2 cells were determined by the transwell migration assay and MTT assay, respectively. Cell cycle and apoptosis of Hep-2 cells were detected by flow cytometry.

**Results:**

The mRNA and protein expression of *14-3-3epsilon *in larynx squamous cell carcinoma (LSCC) tissues were significantly lower than those in clear surgical margin tissues. Statistical analysis showed that the 14-3-3epsilon protein level in metastatic lymph nodes was lower than that in paired tumour tissues. In addition, the protein level of 14-3-3epsilon in stage III or IV tumours was significantly lower than that in stage I or II tumours. Compared with control Hep-2 cells, the percentages of viable cells in the 14-3-3epsilon-GFP and negative control GFP groups were 36.68 ± 14.09% and 71.68 ± 12.10%, respectively. The proportions of S phase were 22.47 ± 3.36%, 28.17 ± 3.97% and 46.15 ± 6.82%, and the apoptotic sub-G1 populations were 1.23 ± 1.02%, 2.92 ± 1.59% and 13.72 ± 3.89% in the control, negative control GFP and 14-3-3epsilon-GFP groups, respectively. The percentages of the apoptotic cells were 0.84 ± 0.25%, 1.08 ± 0.24% and 2.93 ± 0.13% in the control, negative control GFP and 14-3-3epsilon-GFP groups, respectively. The numbers of cells that penetrated the filter membrane in the control, negative control GFP and 14-3-3epsilon-GFP groups were 20.65 ± 1.94, 17.63 ± 1.04 and 9.1 ± 0.24, respectively, indicating significant differences among the different groups.

**Conclusions:**

Decreased expression of *14-3-3epsilon *in LSCC tissues contributes to the initiation and progression of LSCC. *14-3-3epsilon *can promote apoptosis and inhibit the invasiveness of LSCC.

## Background

Squamous cell carcinoma of the head and neck (SCCHN) is considered the sixth most common cancer in the world [1]. More than a half-million new cases of head and neck cancers are reported annually worldwide [2]. Larynx squamous cell carcinoma (LSCC) constitutes almost 2% to 3% of all malignant tumours, representing the second most common malignant neoplasm of the respiratory tract [3]. Each year, around 700 new cases of LSCC in the Netherlands [4] and 10,000 cases in the United States [5] are diagnosed. In China, the incidence of LSCC has been rising gradually, especially in the Northeast. The data mentioned above indicate that laryngeal cancer has become one of the most important cancers impairing human life.

Risk factors such as smoking and alcohol abuse are associated with the development of LSCC [6]. Early laryngeal cancer can usually be managed successfully with either radiotherapy or surgery. Advanced stage cancer often requires a combination of treatment modalities. Depending on tumour stage, the local recurrence rate varies from 10 to 50% [4]. Until now, total laryngectomy or laryngopharyngectomy remains the procedure of choice for advanced stage laryngeal carcinoma around the world [7].

Carcinogenesis involves complex processes including many types of genetic changes, such as the activation of oncogenes and the inactivation of tumour suppressor genes [8]. With the development of molecular biology, there is potential for the use of biomarkers in the diagnosis of LSCC in the future and the results from the study of the molecular mechanisms of LSCC will provide useful biomarkers of LSCC. At present, the biological targets required for diagnosis of LSCC are still unknown.

In our previous study, we screened and identified several proteins, including tyrosine 3-monooxygenase/tryptophan 5-monooxygenase activation protein (14-3-3epsilon), related to DNA methylation in laryngeal carcinoma Hep-2 cells treated with 5-aza-2'-deoxycitydine (5-Aza-CdR). 14-3-3epsilon is one of the mammalian 14-3-3 protein family members that contain a few regions of diversity and have been proposed to interact with more than 200 proteins [9].

14-3-3epsilon is a small acidic protein of about 30 kDa that has the highest homology and is one of the most conserved proteins in organic evolution. 14-3-3epsilon regulates diverse biological processes, including cell cycle control, proliferation, and apoptosis, and plays a significant role in neurogenesis and the formation of malignant tumours. However, the exact function and regulatory mechanism of 14-3-3epsilon in carcinogenesis are not clear. In this study, we explored the role of 14-3-3epsilon in the development and aggression of LSCC by analysing the expression and biological characteristics of *14-3-3epsilon *in LSCC.

## Methods

### Samples

One hundred one cases of LSCC tissues were obtained from patients treated at the Ear, Nose and Throat (E.N.T) Department of the 463 Hospital of PLA of China after receiving their informed consent and the approval of the hospital authorities. None of the patients received radiotherapy or chemotherapy prior to the genetic analysis. The clinical pathological characteristics of the patients were evaluated according to the International Union Against Cancer guidelines. All specimens, which were pathologically primary tumours, included cancerous tissues and matched clear surgical margin tissues typically 4-15 mm in diameter, and 9 cases also contained metastatic lymph node tissues. All specimens were frozen after collection and stored at -80°C immediately. All patients who donated specimens were monitored after the surgery. Among patients treated with total laryngectomy, no recurrent signs were found. Among the patients who underwent partial laryngectomy, local recurrence on the surgical margin of 3 patients has been confirmed. Then, total laryngectomy was performed on these patients and no further neoplasm was found after 12 months follow-up. On the other hand, neck masses have been observed in 12 patients, and these masses exhibited regression after radiotherapy. Because of the negative result from the puncture biopsy and the lack of direct recurrent evidence, we treated these patients as disease-free survivors. Approval for the study was received from the Ethics Committee of China Medical University. Patient information is shown in Table 1.

**Table 1:** The characteristics of the Patients (n = 101).

Characteristic	No. of patients (%)
Age, years	

< 62	51 (50.5)

≥ 62	50 (49.5)

Sex	

Male	81 (80.2)

Female	20 (19.8)

Clinical Stages	

I (T_1_N_0_M_0_)	19 (18.8)

II (T_2_N_0_M_0_)	19 (18.8)

III (T_3_N_0_M_0_, T_1-3_N_1_M_0_)	26 (25.7)

IV (T_4a_N_0-1_M_0_, T_1-4a_N_2_M_0_, T_1-4_N_3_M_0_, T_4b_N_1-3_M_0_, T_1-4_N_1-3_M_1_)	37 (36.7)

metastatic lymph nodes	9 (8.9)

### Semi-quantitative reverse transcription-polymerase chain reaction (RT-PCR)

Total RNA was isolated with Trizol reagent according to the instructions and cDNA was reversibly transcribed from the isolated mRNA using an AMV RNA PCR kit (TaKaRa, China) in line with the standard operating protocol. The upstream primer sequence for *14-3-3epsilon *was 5'-ACG ACG AAA TGG TGG AGT-3', and the downstream sequence was 5'-AGC TGC TGG AAT GAG GTG-3', which were expected to produce a 278-bp DNA fragment. *β-actin *served as an internal control to ensure that an equal amount of mRNA was analysed from each sample. The upstream primer sequence for *β-actin *was 5'-CCA GAT CAT GTT TGA GAC CT-3', and downstream sequence was 5'-TTG AAG GTA GTT TCG TGG AT-3', which were expected to produce a 480-bp DNA fragment. The PCR reaction was performed in a 25-μl system, starting with denaturation at 94°C for 3 min, then 30 cycles of denaturation at 94°C for 30 sec, annealing at 56°C for 45 sec, and extension at 72°C for 45 sec, followed by an extra extension at 72°C for 10 min. The PCR products were separated by 1.2% agarose gel electrophoresis, stained with ethidium bromide and photographed.

### Western blot

For sample preparation, 100 mg of tissue was taken from each sample and ground to a powdery preparation with liquid nitrogen. Twenty micrograms of each sample was lysed by 250 μl of protein extracting fluid (RIPA Lysis Buffer: 50 mM Tris (pH 7.4), 150 mM NaCl, 1% Triton X-100, 1% sodium deoxycholate, 0.1% SDS; PMSF), homogenised for 10 min, incubated in an ice-bath for 1 h, and centrifuged at 12,000 g for 30 min at 4°C. The supernatant was finally collected, and the protein concentration was determined using the BCA protein assay system (Pierce, Rockford, Illinois, USA). Proteins (50 μg/lane) were separated by 12% sodium dodecyl sulphate-polyacrylamide gel electrophoresis (SDS-PAGE) and then transferred to PVDF membranes. After blocking overnight at 4°C with 1× PBS with 0.1% Tween 20 and 5% non-fat milk, the membranes were incubated with 14-3-3epsilon polyclonal antibody (1:800, Santa Cruz, USA) for 3 h at room temperature, washed twice and then incubated again with horseradish peroxidase-conjugated goat anti-rabbit secondary antibody (ZhongShan, China, 1:1,500) for 2 h at room temperature. Immunodetection was performed with chemiluminescence (ECL reagent, Beyotime, China) and the membranes were exposed to film. The image was obtained with a transmission scanner. For quantification, the target proteins were normalised to the internal standard protein β-tubulin by comparing the gray-scale values of 14-3-3epsilon to β-tubulin, which were analysed with the UVP Gelworks ID advanced version 2.5 software (Bio-Rad, USA) [10].

### Construction of 14-3-3epsilon-GFP expression vector

The entire open reading frame of *14-3-3epsilon *complementary DNA (cDNA) was obtained by RT-PCR from mRNA of Hep-2 cells. The forward primer used in the PCR reaction was 5'-ttt AGA TCT tcc gct tcc atc cgt c-3', which included a *Bgl II *site (the capital letters) at the 5' end. The reverse primer was 5'-g tgt ccc tGA ATT Ctc ttg ttg gct tat-3', which contained a *EcoR I *site (the capital letters) at the 5' end. The PCR product covered the initiation codon and its flanking sequences. The PCR reaction was performed in a 50-μl reaction system, starting with denaturation at 94°C for 3 min, then 35 cycles of denaturation at 94°C for 30 sec, annealing at 60°C for 45 sec, and extension at 72°C for 1 min, followed by an extra extension at 72°C for 10 min. The amplified fragments were digested with *Bgl II *and *EcoR I *and cloned into pEGFP-C1 plasmids (BD, USA). The 14-3-3epsilon-GFP expression vector was verified by *Bgl II-EcoR I *digestion and DNA sequencing. *14-3-3epsilon *was expressed by fusion to the C-terminus of EGFP.

### Cell culture and Transient Transfection

The Hep-2 (Human laryngeal carcinoma) cell line was purchased from Cell Biology Institute of Shanghai, Chinese Academy of Science and originated from a metastatic epidermoid carcinoma of the larynx [11]. The cells were maintained in RPMI-1640 supplemented with 10% foetal bovine serum (FBS), 100 U/mL penicillin and 100 μg/mL streptomycin at 37°C in a humidified atmosphere of 5% CO_2 _and 95% air. Upon reaching 60%-70% confluence, the cells were seeded overnight at a density of 1 × 10^5 ^cells per well in six-well plates. 14-3-3epsilon-GFP vectors and pEGFP-C1 vectors (as a negative control) were then transfected into Hep-2 cells using Lipofectamine 2000 (Invitrogen, USA) following the manufacturer's instructions. After 24 h of transfection, the effectiveness of transfection was observed and detected by fluorescence microscopy and RT-PCR, respectively.

### Cell viability assay

After being seeded for 24 h in a 96-well plate, Hep-2 cells (1 × 10^4 ^cells/well) were transfected with GFP and 14-3-3epsilon-GFP for 48 h in 3 parallel wells each, with untransfected cells serving as a control. At 48 h, 10 μl of MTT solution (5 mg/mL) was added to each well and incubated for a further 4 h. The medium was removed and 200 μl of DMSO was added to each well and then vibrated for 10 min. Absorbance (*A*) at 490 nm was measured using a microplate reader. The percentage of viable cells was calculated as follows: (*A *of experimental group/*A *of control group) × 100%. Data were indicated as the means of the triplicate determinations [10].

### Cell cycle assay

After incubation at 37°C for 48 h, cells were harvested in cold PBS and washed once with 1× PBS, fixed in 70% EtOH, and stored at 4°C for 24 h. The fixed cells were washed with 1× PBS once, suspended in 400 μl of 50 mg/ml PI staining reagent (Sigma, USA), and then incubated in the dark for 30 min. The distribution and quantitation of cells in cell cycle distribution were detected by flow cytometry [10].

### Apoptosis assay

The apoptotic rates were analysed by flow cytometry using an annexin V-PE/7-AAD Kit (KeyGEN, China). Staining was performed according to the manufacturer's instructions, and flow cytometry was conducted on a FACSCalibur (Becton Dickinson, Mountain View, NJ, USA). Cells that were both annexin V-PE and 7-AAD negative were considered viable cells. Cells that were annexin V-PE positive and 7-AAD negative indicated early apoptotic cells. Cells that were both annexin V-PE and 7-AAD positive represented late apoptotic cells [10].

### Transwell chamber invasion assay

Twenty-four-well invasion chambers were obtained from Costar. Hep-2 cells transfected with negative control GFP and 14-3-3epsilon-GFP were detached from the tissue culture plates, washed, resuspended in conditioned medium (2 × 10^5 ^cells/ml), and added to the upper compartment of the invasion chamber. Five hundred microlitres of conditioned medium was added to the lower compartment of the invasion chamber. The invasion chambers were then incubated at 37°C for 24 h. After incubation, the inserts and cells on the upper side of the filter were removed. The filters were fixed, mounted, and stained according to the manufacturer's instructions. The cells that invaded to the underside of the filter were counted. Each experiment was repeated three times. The values obtained were calculated by averaging the total number of cells from triplicate determinations [12].

### Statistical analysis

Statistical analysis was performed using SPSS 17.0. All data were expressed as means ± standard error of the mean (SEM). The comparisons between mRNA and protein expression levels in one group between tumour and matched clear surgical margin tissues and in the other group between tumour and metastatic lymph nodes were made by the paired sample t-test for parametric analysis or Wilcoxon signed rank test for nonparametric analysis. Comparisons related to age or sex in clinical characteristics were made by the Mann-Whiney U test. The differences between experimental and control groups and among gene expression levels related to clinical stages were analysed by one-way analysis of variance (ANOVA). The correlation between mRNA and protein levels was analysed by Spearman rank correlation. Statistical significance was assumed for a two-tailed p < 0.05.

## Results

### Reduced expression of 14-3-3epsilon in LSCC

**Figure 1:**
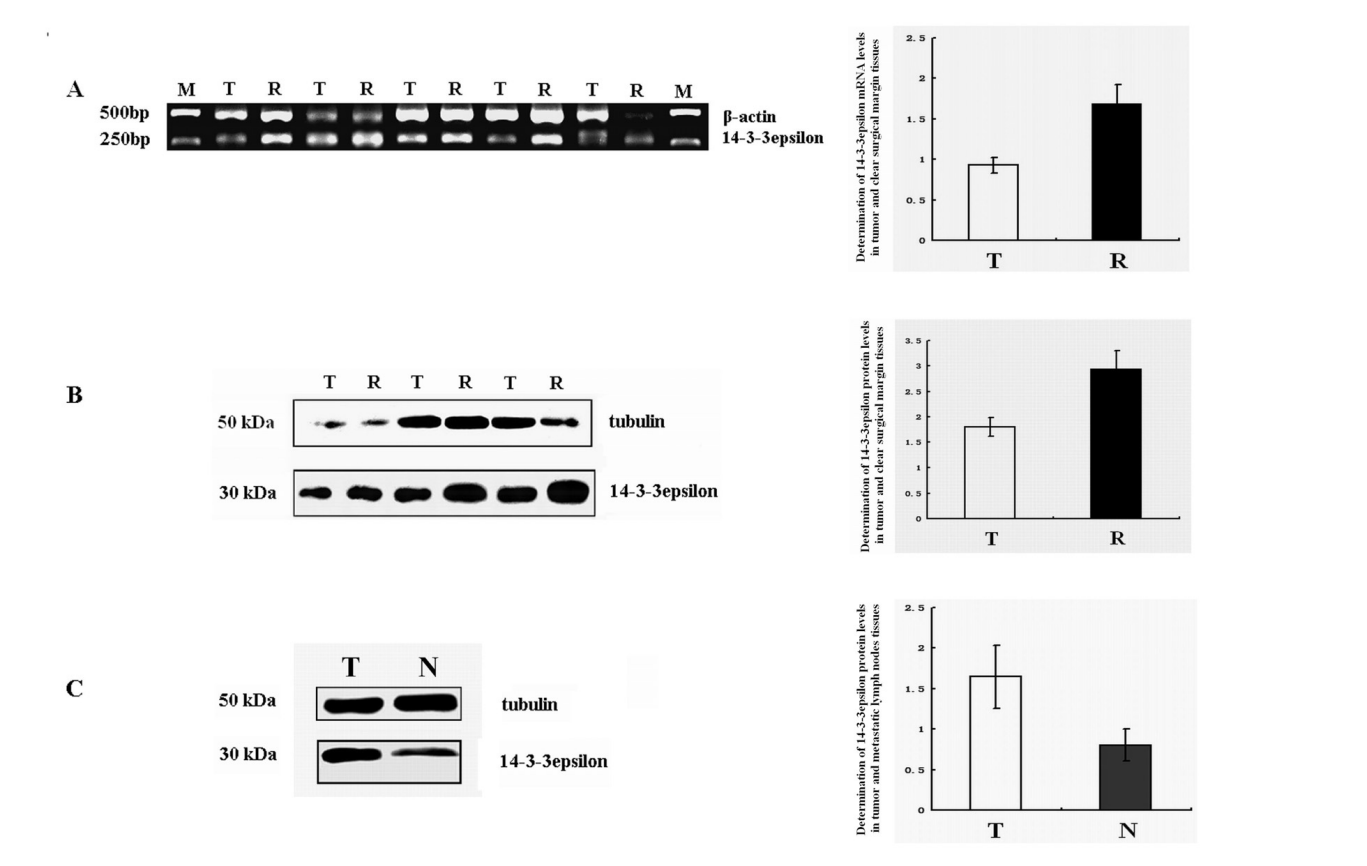
**Expression of *14-3-3epsilon *in LSCC**. **A**. *14-3-3epsilon *mRNA levels in LSCC. **B **and **C**. 14-3-3epsilon protein levels in LSCC. M, DNA marker; T, tumour; R, clear surgical margin; N, metastatic lymph node tissues.

*14-3-3epsilon *mRNA levels in 72 of the 101 cases of LSCC and matched clear surgical margin tissues were evaluated by RT-PCR. The result showed that the *14-3-3epsilon *mRNA expression level in LSCC tissues was significantly lower than that in clear surgical margin tissues (p = 0.008, Figure 1A and Table 2). 14-3-3epsilon protein levels were detected in all 101 cases of LSCC tissues and matched clear surgical margin tissues and in all 9 cases of metastatic lymph node tissues by western blotting. The 14-3-3epsilon protein expression level in LSCC tissues was significantly lower than that in clear surgical margin tissues (p = 0.002, Figure 1B and Table 2). However, there was no significant correlation between mRNA levels and protein levels (data not shown). The 14-3-3epsilon protein expression level in the metastatic lymph nodes was also lower than that in matched LSCC tissues (p = 0.037, Figure 1C and Table 3).

**Table 2:** Analysis of *14-3-3epsilon *mRNA and protein levels in tumour and clear surgical margin tissues.^a^

Tissues	*14-3-3epsilon *mRNA	14-3-3epsilon protein
Tumour	0.9284 ± 0.0938	1.8003 ± 0.1867

Clear surgical margin	1.6822 ± 0.2447	2.9328 ± 0.3675

p-value	0.008	0.002

^a^Gray-scale ratio, mean ± SEM,	n = 72	n = 101

**Table 3:** Analysis of *14-3-3epsilon *protein levels in tumour and metastatic lymph node tissues.^a^

Tissues	14-3-3epsilon protein
Tumour	1.6460 ± 0.3897

metastatic lymph nodes	0.8040 ± 0.2006

p-value	0.037

^a^Gray-scale ratio, mean ± SEM	n = 9

We assessed the *14-3-3epsilon *expression levels with respect to clinical characteristics (age, sex and the clinical stages). No differences were identified in protein and mRNA levels of *14-3-3epsilon *with respect to patient age and sex (data not shown). There was no difference in mRNA levels with respect to patient clinical stage (Table 4). However, the protein expression level of 14-3-3epsilon in stage III or IV tumours was significantly lower than that in any stage I or II tumours (p < 0.001, Table 4).

**Table 4:** Analysis of the relationship between *14-3-3epsilon *and Clinical Stages.^a^

Clinical stages	*14-3-3epsilon *mRNA	14-3-3epsilon protein
I	1.1288 ± 0.2341	3.0582 ± 0.5554

II	0.9747 ± 0.1976	2.8348 ± 0.5532

III	0.6006 ± 0.1414	1.1029 ± 0.1744

IV	0.9661 ± 0.1609	1.1132 ± 0.1763

p-value	0.342	< 0.001

^a^Gray-scale ratio, mean ± SEM

### Decreased proliferation of Hep-2 cells transfected with *14-3-3epsilon*

**Figure 2:**
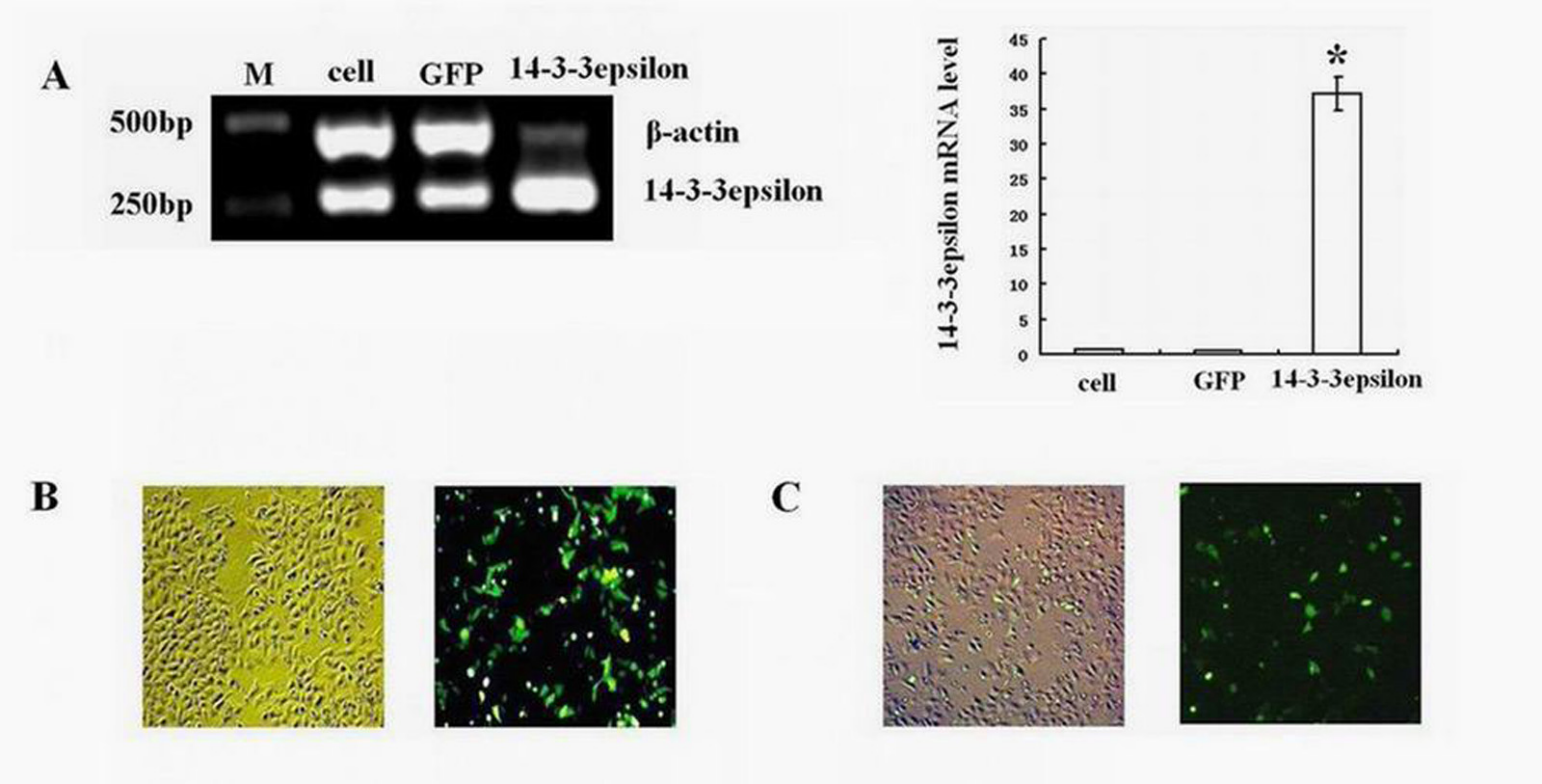
**Transfection of *14-3-3epsilon *in Hep-2 cells**. **A**. mRNA levels of *14-3-3epsilon *in Hep-2 cells transfected with 14-3-3epsilon-GFP. **B**. GFP as a negative control was revealed by contrast and fluorescence microscopy under the same phase. **C**. Transfected 14-3-3epsilon-GFP was revealed by contrast and fluorescence microscopy under the same phase. Lanes: M, DNA marker; cell, control cells; GFP, negative control of GFP; 14-3-3epsilon, transfected 14-3-3epsilon-GFP (*p < 0.05).

**Figure 3:**
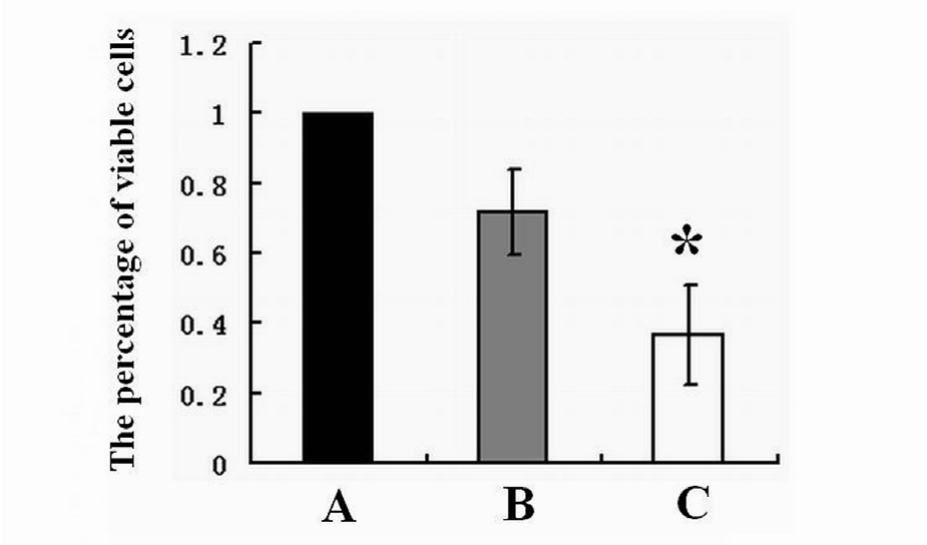
**The proliferation of Hep-2 cells**. **A**. The percentage of viable Hep-2 cells before transfection. **B**. The percentage of viable Hep-2 cells after transfection with GFP. **C**. The percentage of viable Hep-2 cells after transfection with 14-3-3epsilon-GFP (*p < 0.05).

Hep-2 cells were transfected with 14-3-3epsilon-GFP and GFP expression vectors. Twenty-four hours after transfection, Hep-2 cells in the 14-3-3epsilon-GFP group showed a significant increase in *14-3-3epsilon *expression at both mRNA (Figure 2A, p < 0.05) and protein levels compared to those in the blank and negative groups (Figure 2B and 2C). As shown in Figure 3, the percentages of viable cells in 14-3-3epsilon-GFP and negative control GFP groups compared to that in blank group were 36.68 ± 14.09% and 71.68 ± 12.10%, respectively, which indicated that the growth of Hep-2 cells decreased significantly after 14-3-3epsilon-GFP transfection (p = 0.008).

### S phase arrest of Hep-2 cells with overexpression of *14-3-3epsilon*

**Figure 4:**
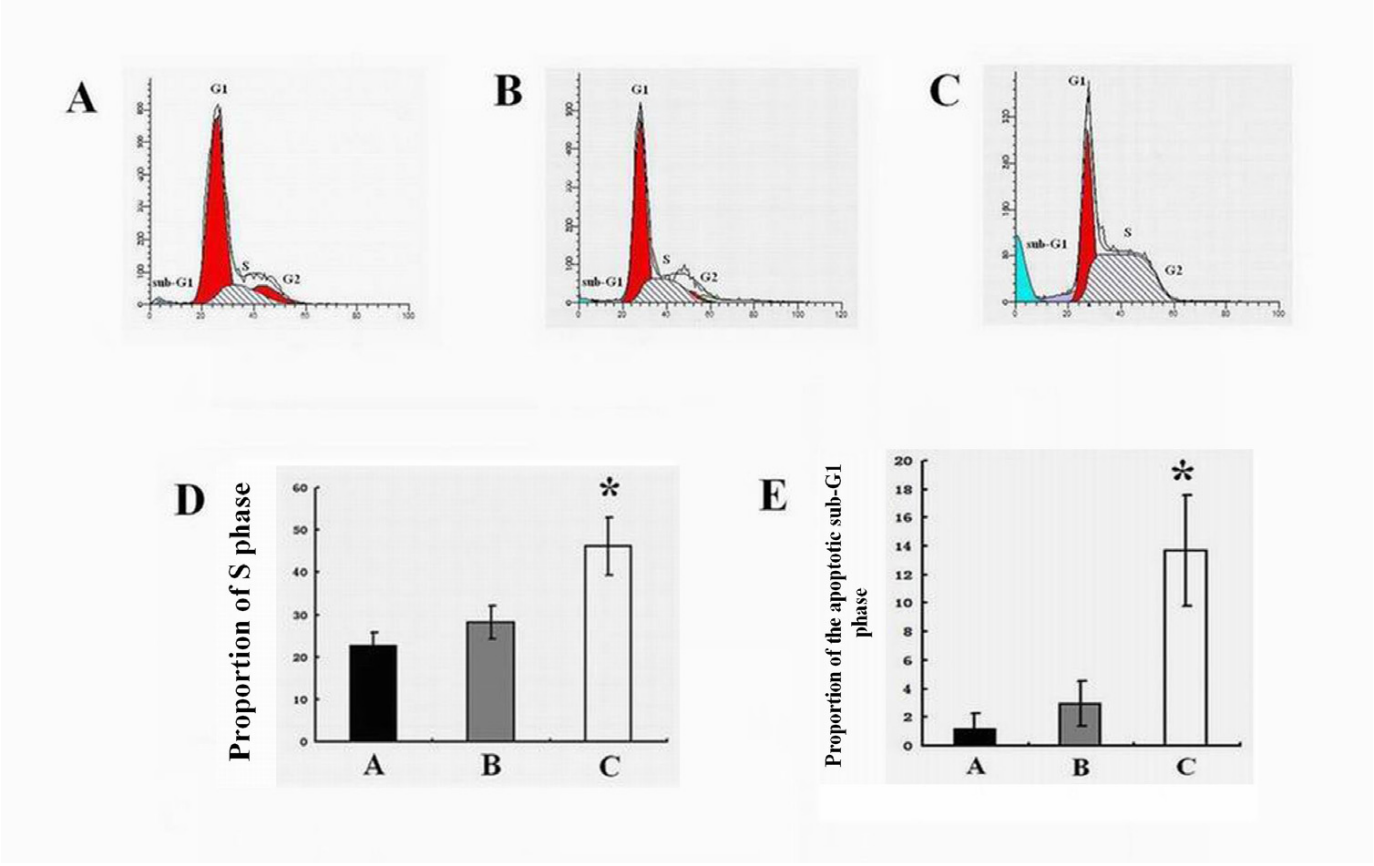
**Cell cycle control of Hep-2 cells**. **A**. Cell cycle analysis of Hep-2 cells before transfection. **B**. Cell cycle analysis of Hep-2 cells transfected with GFP. **C**. Cell cycle analysis of Hep-2 transfected with 14-3-3epsilon-GFP. **D**. The percentages of cells in S phase from the blank, GFP and 14-3-3epsilon-GFP groups, respectively (*p < 0.05). **E**. The proportions of cells in apoptotic sub-G1 phase from the blank, GFP and 14-3-3epsilon-GFP groups, respectively (*p < 0.05).

Compared with the blank control (Figure 4A) and negative control GFP groups (Figure 4B), a significant accumulation of cells in the S phase of the cell cycle and a concomitant increase in the apoptotic sub-G1 population were noted in the 14-3-3epsilon-GFP group (Figure 4C). In the control, negative and 14-3-3epsilon-GFP groups, the proportions of S phase cells were 22.47 ± 3.36%, 28.17 ± 3.97% and 46.15 ± 6.82%, respectively (p = 0.034, Figure 4D) and the apoptotic sub-G1 population significantly increased from 1.23 ± 1.02% and 2.92 ± 1.59% in the control and negative groups, respectively, to 13.72 ± 3.89% in the 14-3-3epsilon-GFP group (p = 0.024, Figure 4E).

### Increased apoptosis of Hep-2 cells transfected with *14-3-3epsilon*

**Figure 5:**
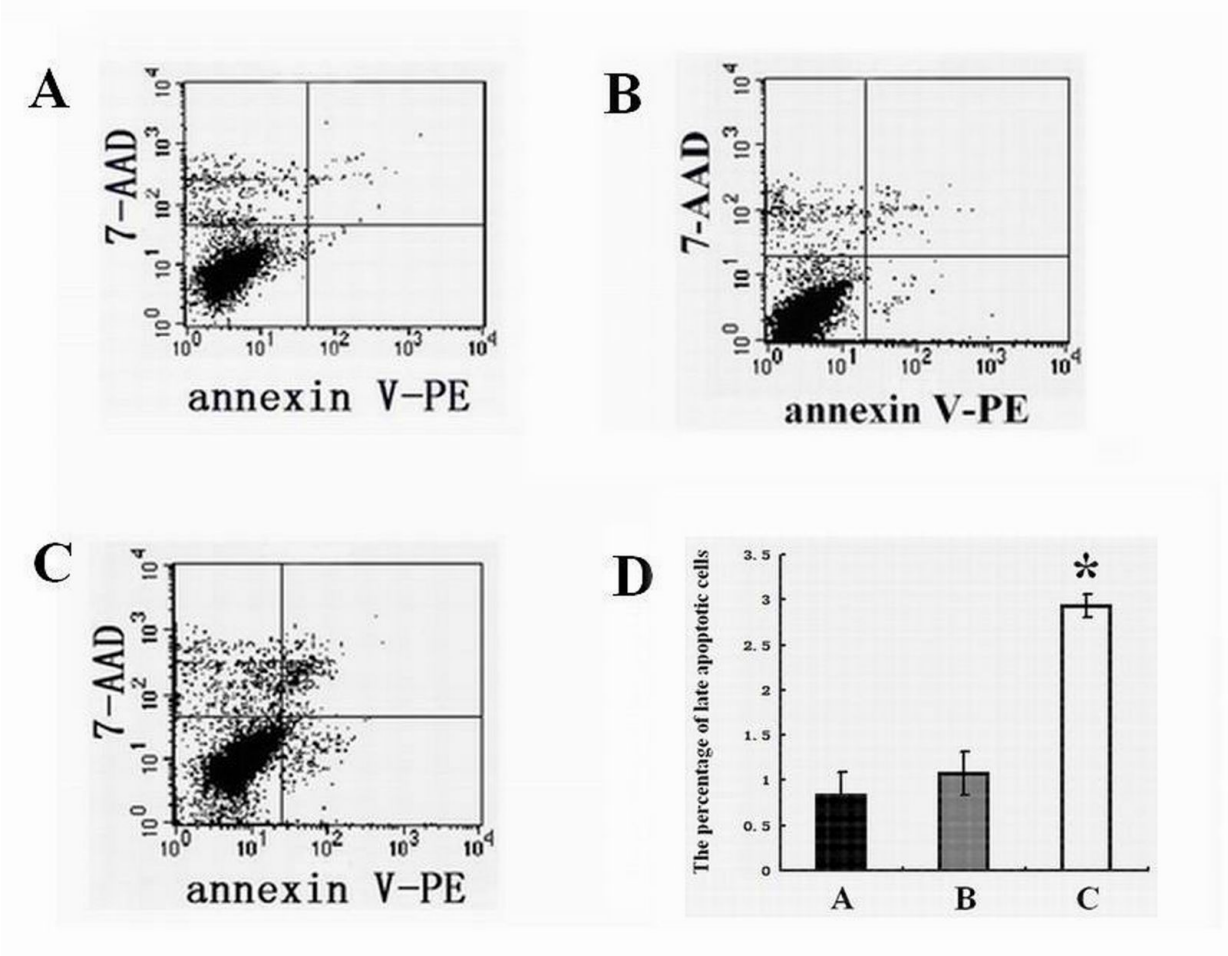
**Late apoptosis of Hep-2 cells**. **A**. Apoptotic chart of Hep-2 cells before transfection. **B**. Apoptotic chart of Hep-2 cells transfected with GFP. **C**. Apoptotic chart of Hep-2 transfected with 14-3-3epsilon-GFP. **D**. The percentages of late apoptotic cells in the blank, GFP and 14-3-3epsilon-GFP groups, respectively (*p < 0.05).

Compared with blank control (Figure 5A) and negative control GFP groups (Figure 5B), the number of the late apoptotic cells significantly increased in 14-3-3epsilon-GFP group cells (Figure 5C). The percentages of the apoptotic cells in control, negative and 14-3-3epsilon-GFP groups were 0.84 ± 0.25%, 1.08 ± 0.24% and 2.93 ± 0.13%, respectively, which showed significant differences among the different groups (p < 0.001, Figure 5D).

### Decreased invasiveness in Hep-2 cells transfected with 14-3-3epsilon

**Figure 6:**
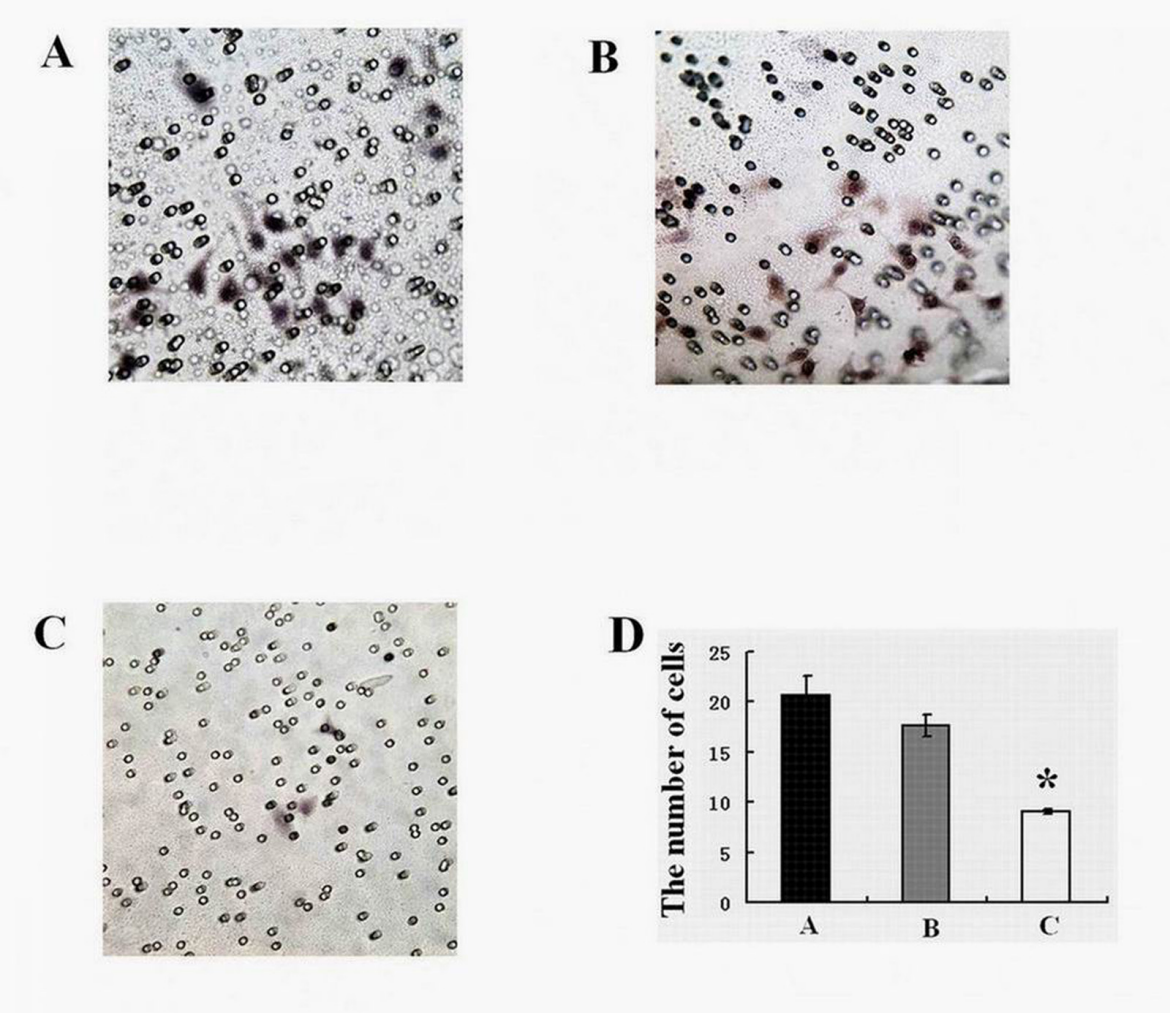
**The invasive ability of Hep-2 cells**. **A**. Invasive chart of Hep-2 cells before transfection. **B**. Invasive chart of Hep-2 cells transfected with GFP. **C**. Invasive chart of Hep-2 transfected with 14-3-3epsilon-GFP. **D**. The transmembrane cells in the blank, GFP and 14-3-3epsilon-GFP groups, respectively (*p < 0.05).

Compared to those in the blank control (Figure 6A) and negative control GFP groups (Figure 6B), the number of cells migrating across the membranes in the 14-3-3epsilon-GFP group (Figure 6C) decreased dramatically. The numbers of cells penetrating the filter membrane in the control, negative and 14-3-3epsilon-GFP groups were 20.65 ± 1.94, 17.63 ± 1.04 and 9.1 ± 0.24, respectively, which showed significant differences among the different groups (p < 0.001, Figure 6D).

## Discussion

14-3-3epsilon is a member of the 14-3-3 protein family comprising a series of highly conserved small acidic proteins of about 29-33 kDa. 14-3-3 proteins, which were originally identified as brain-specific, are present in a wide range of organisms and tissues. These proteins normally exist as homo- or heterodimers. The 14-3-3 dimer serves as an adaptor that couples with target proteins to stimulate protein-protein interactions, regulate the subcellular localisation of proteins and activate or inhibit associated enzymes. The binding between a 14-3-3 protein and its substrate is generally triggered by phosphorylation of the target protein at specific Ser/Thr residues. In this manner, 14-3-3 proteins are key regulators of phosphoprotein targets within a variety of processes, such as the regulation of cell signalling, cell cycle progression, signal transduction, intracellular trafficking/targeting, cytoskeletal structure, transcription and apoptosis [13-17].

14-3-3epsilon also regulates a wide range of biological processes. Abnormal expression of *14-3-3epsilon *has been found in several types of cancers. Low expression of *14-3-3epsilon *occurred in lung cancers [18] and medulloblastoma [19]. High expression of *14-3-3epsilon *was detected in subependymomas [20]. At present, there are few clues about the role and molecular mechanism of *14-3-3epsilon *in carcinogenesis, and no information of *14-3-3epsilon *related to LSCC has been reported.

In this study, the general tendency of *14-3-3epsilon *mRNA and protein expression levels was consistent, which means that *14-3-3epsilon *expression at both mRNA and protein levels was down-regulated in LSCC compared with those in the clear surgical margin tissues. However, there was no significant correlation between mRNA and protein levels in LSCC, which could be caused by mechanisms such as inhibition of microRNAs in translation. There was also no relationship between *14-3-3epsilon *expression levels and sex or age in patients suffering with LSCC, which shows that sex and age do not affect the expression levels of *14-3-3epsilon *in LSCC. Although there was no significant difference between 14-3-3epsilon protein levels at stage III and IV or stage I and II, a significant difference between 14-3-3epsilon protein levels at stage III or IV and those at stage I or II in LSCC was observed, which indicates that the 14-3-3epsilon protein may be useful in identifying metastatic or locally advanced LSCC tumours.

14-3-3epsilon plays a role in the G2 DNA damage checkpoint response, which results in G2 phase arrest in different cancer cell lines due to inhibition of cdc25C [18,21,22]. However, our study showed that the growth of Hep-2 cells overexpressing 14-3-3epsilon was inhibited and these cells were only halted in S phase, which indicates that the low proliferation of Hep-2 cells transfected with 14-3-3epsilon-GFP originates partly from S phase arrest. The molecular mechanism of how the arrest of Hep-2 cells in S phase is affected by 14-3-3epsilon will be an interesting area of future study.

Some studies show that 14-3-3epsilon, an inhibitor of apoptosis proteins, prevents apoptosis progression by inhibiting the activities of pro-apoptotic proteins such as Bad and Bax [23-26]. However, in the present study, our results from both apoptosis and cell cycle assays showed that the number of apoptotic cells in the 14-3-3epsilon-GFP group increased, which indicates that *14-3-3epsilon *can promote apoptosis. We speculate that the increased apoptosis in Hep-2 cells transfected with 14-3-3epsilon could also lead to a reduction of cell numbers. Meanwhile, the down-regulation of *14-3-3epsilon *detected in LSCC in the study perhaps indicates a role for *14-3-3epsilon *in the development of LSCC by inhibiting apoptosis.

Normal tissue invasion and metastasis are hallmarks of malignant tumours. Cancer cell metastasis to distant organs is the major cause of death in almost all forms of cancer. Metastasis is a multi-step process, and the initial step is the invasion of surrounding tissues by cancer cells. Inhibition of the invasion and metastasis pathways of tumour cells could provide new treatment alternatives for cancer patients [27,28]. Tak et al. found that *14-3-3epsilon *inhibits cell migration in HeLa cells by interacting with MAPK-activated protein kinase 5 (MK5) [29]. Our present study showed that *14-3-3epsilon *displayed lower expression in the metastatic lymph nodes compared to that in cancer tissues and 14-3-3epsilon protein levels were significantly lower in stage III or IV compared to those in stage I or II, which implies that *14-3-3epsilon *might inhibit the metastasis of LSCC. Additionally, our transwell result supports this conclusion. The results from apoptosis, cell cycle and cell viability assays combined with those mentioned above in the study implies that the lower expression of *14-3-3epsilon *that results in decreased apoptosis and high proliferation could contribute to invasion and aggression of LSCC.

According to the achieved results in the present study, *14-3-3epsilon *could be a useful parameter for diagnosing LSCC. It could also be used as a molecular marker to determine clinical staging. Meanwhile, 14-3-3epsilon may be a potential target of a new drug that can control the initiation and progression of LSCC effectively.

## Conclusions

Decreased expression of *14-3-3epsilon *in LSCC tissues contributes to the initiation and progression of LSCC. *14-3-3epsilon *can promote apoptosis and inhibit the invasiveness of LSCC. The exact molecular mechanisms of *14-3-3epsilon *in apoptosis and aggression of LSCC require further investigation.

## Competing interests

The authors declare that they have no competing interests.

## Authors' contributions

XHC participated in the design of the study, carried out the mRNA and protein experiments, expression vector construction, cell viability, cell cycle, apoptosis, and invasion assays, statistical and data analysis and drafted the manuscript. HC carried out the cell culture and transient transfection assays and interpreted and revised the manuscript critically. ZMX collected clinical data, participated in the interpretation of data and helped to draft the manuscript. CS participated in gene expression analysis and revised the manuscript critically. KLS was involved in the project design and manuscript revising. WNF participated in the design of the study, mRNA and protein experiments, and statistical and data analysis and helped to draft the manuscript. All authors read and approved the final manuscript.

## Pre-publication history

The pre-publication history for this paper can be accessed here:

http://www.biomedcentral.com/1471-2407/10/306/prepub
